# Obesity-Related Kidney Disease in Bariatric Surgery Candidates

**DOI:** 10.1007/s11695-024-07602-w

**Published:** 2024-12-05

**Authors:** Pedro Reis Pereira, Manuela Almeida, Patrícia Braga, João Pereira, Sofia Pereira, Mário Nora, Marta Guimarães, Jorge Malheiro, La Salete Martins, Mariana P. Monteiro, Anabela Rodrigues

**Affiliations:** 1 Department of Nephrology, Unidade Local de Saúde de Santo António, (ULS Santo António), Porto, Portugal; 2 Unit for Multidisciplinary Research in Biomedicine (UMIB), School of Medicine and Biomedical Sciences (ICBAS), University of Porto, Porto, Portugal; 3 ITR - Laboratory for Integrative and Translational Research in Population Health, Porto, Portugal; 4 General Surgery Department and CRI for the surgical Treatment of Obesity and Metabolic Diseases, ULS Entre o Douro e Vouga, Santa Maria da Feira, Portugal

**Keywords:** Fatty kidney, Hyperfiltration, Living kidney donation, Proteinuria, Obesity-related glomerulopathy

## Abstract

**Background:**

Obesity has a negative impact in kidney health. However, the hallmarks of kidney dysfunction in bariatric surgery candidates are poorly characterized. To address this knowledge gap, we used a propensity score-matched analysis to compare kidney lesion biomarkers in bariatric surgery candidates and living kidney donors.

**Methods:**

Bariatric surgery candidates attending a single center for obesity treatment were pair-matched for sex and age to potential living kidney transplant donors (PLKD) using a 1:1 nearest-neighbor approach (*N* = 400, *n* = 200/group). A 24-h urine collection was used to analyze proteinuria and creatinine clearance.

**Results:**

Patients with obesity (PWO) had higher creatinine clearance when compared to PLKD (143.35 ± 45.50 mL/min vs 133.99 ± 39.06 mL/min, *p* = 0.03), which was underestimated when correction for body surface area (BSA) was used (creatinine clearance corrected for BSA of 115.25 ± 33.63 mL/min/1.73 m^2^ in PWO vs 135.47 ± 35.56 mL/min/1.73 m^2^ in PLKD). Proteinuria was also higher in PWO compared to PLKD (139.82 ± 353.258 mg/day vs 136.35 ± 62.24 mg/day, *p* < 0.0001). Regression analysis showed that creatinine clearance was strongly correlated with proteinuria in PWO (HR 1.522, *p* = 0.005), but it was less evident in PLKD (HR 0.376, *p* = 0.001).

**Conclusion:**

Hyperfiltration and disproportionate proteinuria are frequent in patients with obesity. Since hyperfiltration can be underestimated by adjusting creatinine clearance for BSA, this should not be used when evaluating kidney function in bariatric surgery candidates.

## Introduction

Obesity has significant repercussions on kidney health [1]. Obesity leads to the progression of chronic kidney disease (CKD) irrespective of the underlying etiology and causes specific kidney disorders, such as obesity-related glomerulopathy [2, 3].

Hyperfiltration, frequently observed in the setting of obesity, is believed to play a significant role in the development of obesity-related kidney disorders [2]. Other possible kidney lesion pathways include tubulointerstitial dysfunction with excess tubular sodium reabsorption, overactivation of the renin–angiotensin–aldosterone system, and renal sympathetic nervous system [4–8]. Additionally, adipose tissue-derived hormones [9] and pro-inflammatory adipokines, such as leptin, resistin, fetuin-A, angiopoietins, vascular endothelial growth factor (VEGF), cathepsins, cystatin-C, and reactive oxygen species (ROS), can also have a negative impact on kidney function [10]. Free fatty acids (FFAs) derived from perirenal fat accumulation have been also shown to damage the kidney cortex and tubules [8, 11]. Altogether, these factors may trigger an adaptative systemic response favoring ectopic lipid accumulation and fibrogenesis that results in tubuloglomerular injury [2, 12–15], similar to what is observed in the liver [16].

Evaluating kidney function in patients with obesity (PWO) can be challenging. To account for body size differences, estimated glomerular filtration rate (eGFR) formulas usually correct the glomerular filtration value for body surface area (BSA). However, since eGFR does not increase in parallel with adiposity, being rather the end result of the increase of single nephron filtration, indexing creatinine clearance for BSA ends up concealing hyperfiltration in patients with obesity [17, 18]. This has previously been demonstrated in a study comparing the performance of 56 formulas based on creatinine and/or cystatin C with measured GFR [19], suggesting that BSA indexing should generally be abandoned in PWO.

Living donor kidney transplantation is the best available treatment for end-stage CKD [20]. Potential living kidney donor (PLKD) candidates are routinely screened for health conditions that are contraindications for kidney donation by living individuals[21]. This process allows to exclude individuals found to have overt kidney function abnormalities among an overall healthy population.

In this study, we sought to compare kidney function markers of individuals with or without obesity recruited among candidates for bariatric surgery or living kidney donation. Because sex and age are known to be important determinants of both kidney function and body composition [22, 23], we used propensity matching to identify the individuals included in the analysis in order to minimize the differences in baseline characteristics between groups.

## Materials and Methods

### Patients

This study included patients with obesity (PWO) recruited among bariatric surgery candidates attending a center for surgical treatment of obesity between 2019 and 2022 and another group without obesity recruited among individuals attending a center for evaluation of living kidney transplant donors (PLKD) candidates between 2008 and 2019.

In the first group, patients were adults eligible for surgical treatment of obesity with body mass index (BMI) higher than 40 kg/m^2^ or with BMI higher than 35 kg/m^2^ in the presence of obesity-related comorbidities. In the second group, subjects had been validated as living kidney transplant donors. Criteria to be admitted as a living kidney donor followed the Kidney Disease: Improving Global Outcomes (KDIGO) 2017 clinical practice guideline for evaluating living donor candidates [24] and the British Guidelines for Living Donor Evaluation [25], which are thoroughly described elsewhere [21]; in particular, obesity defined by a BMI greater than 30 kg/m^2^ is an exclusion criterion. In addition, the concomitant diagnosis of any type of diabetes or prediabetes under treatment with metformin, neoplastic diseases, or acute or chronic inflammatory conditions was pre-established exclusion criteria for both study groups. Donors with proteinuria were further evaluated for glomerular pathology and those with confirmed proteinuria over 300 mg/day were also excluded. Normal glycemic status was defined as a glycated hemoglobin level under 5.7% in the absence of any antidiabetic medication; prediabetes was defined as a glycated hemoglobin level between 5.7 and 6.5% and no antidiabetic drugs other than metformin; diabetes was defined as a glycated hemoglobin level greater than 6.5% or treatment with two or more different anti-diabetic drug classes, regardless of the glycated hemoglobin levels.

### Data Acquisition

Data concerning age, gender, body weight, BMI, complete blood count, serum creatinine, uric acid levels, fasting glucose, hemoglobin, lipid profile (total cholesterol, LDL cholesterol, HDL cholesterol, triglycerides), urinalysis, proteinuria, and indexed and non-indexed creatinine clearance based on a 24-h urine collection were acquired for each patient. CKD-EPI 2021 Creatinine was used to calculate the estimated glomerular filtration rate (eGFR) [26].

The study participants were instructed to collect a 24-h urine on two consecutive days before the blood sampling. Individuals were given oral and written instructions on how to perform a 24-h urine collection. Urinary protein and creatinine concentrations were measured in each collection. The 24-h creatinine clearance rate was calculated using the measured serum creatinine concentration and the urine creatinine concentration of the 24-h urine; measurements were calculated both in body surface area (BSA)-indexed and non-indexed forms [27].

Participants were categorized into clinically significant levels of proteinuria (higher or lower than 150 mg/24 h) as defined by the KDIGO guidelines [28]. A threshold of creatinine clearance of 140 mL/min was used to classify patients as having significant hyperfiltration [17].

The study protocol was reviewed and approved by the ethical committees and institutional review boards of both hospital institutions (approvals no. CA-014/20-Ot_MP/CC and 147–21(119-DEFI/122-CE) in accordance with the recommendations of the Declaration of Helsinki and European Data Protection Regulations.

### Propensity Matching

The XLSTAT extension of Microsoft Excel was used to perform a propensity matching between members of both groups according to sex and age. The original data set had 238 individuals in the PWO group and 365 individuals in the PLKD group. One-to-one matching was performed using the Mahalanobis distance technique.

### Statistical Analysis

All data presented are expressed as mean ± standard deviation (SD), unless otherwise specified. The Shapiro–Wilk test was used to determine the normality of the groups. A comparison of independent groups was carried out by using either an unpaired *t*‐test or a Mann–Whitney *U* test, depending on the normality. To compare 2 or more nominal variables, we used a *χ*2 test. To assess relative risk increase/adjusted odds ratios a linear or a binary logistic regression, depending on the variable’s type, was employed using SPSS version 28.0, either by using single or combined variables. The prediction power of different parameters was evaluated using the receiver operating characteristic (ROC) curve. The area under the ROC curve (AUC) was used to measure how well a marker can predict outcome measures. Based on the AUC, the test was considered excellent between 0.90 and 1.00, good between 0.80 and 0.90, fair between 0.70 and 0.80, and poor between 0.60 and 0.70, and the test was considered to have failed if the value was below 0.60. A *p*‐value < 0.05 was considered statistically significant. Statistical analysis was carried out using GraphPad (Prism; Version 8.0.1) and SPSS (IBM; Version 28.0) for Windows.

## Results

Propensity matching returned 200 individuals in each study group of PWO and PLKD, yielding a final population of 400 individuals. Clinical and biochemical characteristics of the participants are depicted in Table 1. Variables related to kidney function are presented in Table 2. BMI was significantly different between groups (41.70 ± 5.32 kg/m^2^ in PWO vs 24.91 ± 3.32 kg/m^2^ in PLKD; *p* < 0.0001) (Table 1).

**Table 1:** Clinical and biochemical characteristics in each study group. Results are presented as mean ± standard deviation (SD)

	PWO	PLKD	*p*-value
BMI (kg/m^2^)	41.70 ± 5.32	24.91 ± 3.32	< 0.0001
Age (years)	41.93 ± 11.40	43.81 ± 10.18	0.084
Female, *n* (%)	161 (80.5)	156 (78.0)	0.622
Uric acid (mg/dL)	5.59 ± 1.32	4.21 ± 1.22	< 0.0001
Glucose (mg/dL)	92.20 ± 11.65	84.59 ± 8.52	< 0.0001
Hemoglobin (g/dL)	13.81 ± 1.15	13.72 ± 1.20	0.20
Total cholesterol (mg/dL)	193.82 ± 36.07	189.48 ± 37.83	0.31
Triglycerides (mg/dL)	125.17 ± 62.48	93.40 ± 50.45	< 0.0001
HDL cholesterol (mg/dL)	49.13 ± 10.91	62.98 ± 16.17	< 0.0001
LDL cholesterol (mg/dL)	133.22 ± 36.22	109.81 ± 31.74	< 0.0001
Lipid-lowering therapy, *n* (%)	18 (9.0)	20 (10.0)	0.864
ACEi/ARB, *n* (%)	25 (12.5)	22 (11.0)	0.756

Abbreviations: *PWO* patient with obesity, *PLKD* potential living kidney donors, *BMI* body mass index, *HDL* high-density lipoprotein, *LDL* low-density lipoprotein, *ACEi* angiotensin converting enzyme inhibitors, *ARB* angiotensin receptor blockers

**Table 2:** Kidney function variables in each study group. Results are presented as mean ± standard deviation (SD)

	PWO	PLKD	*p*-value
Serum creatinine (mg/dL)	0.76 ± 0.13	0.72 ± 0.15	< 0.0001
Proteinuria (mg/day)	139.82 ± 353.26	136.35 ± 62.24	< 0.0001
Proteinuria ≥ 150 mg/day, *n* (%)	44 (22)	77 (38.5)	0.0005
CrCl/BSA (mL/min)	115.25 ± 33.63	135.47 ± 35.56	< 0.0001
CrCl/BSA ≥ 140 mL/min, *n* (%)	41 (20.5)	75 (37.5)	0.0003
CrCl (mL/min)	143.35 ± 45.50	133.99 ± 39.06	0.0305
ClCr ≥ 140 mL/min, *n* (%)	99 (49.5)	78 (39)	0.0439
eGFR (mL/min/1.73 m^2^)	105.43 ± 14.09	106.49 ± 13.63	0.2844
eGFR ≥ 100 mL/min/1.73 m^2^, *n* (%)	138 (69)	149 (74.5)	0.267

Abbreviations: *PWO* patient with obesity, *PLKD* potential living kidney donors, *CrCl* creatinine clearance, *BSA* body surface area, *eGFR* estimated glomerular filtration rate

Average serum creatinine was not significantly different between groups. Creatinine clearance was higher than 140 mL/min in 99 patients (49.5%) in the group of PWO and only in 78 patients (39%) in the group of PLKD (*p* < 0.05). Adjustment of creatinine clearance to BSA underestimated hyperfiltration in the PWO group. Estimation of glomerular filtration rate (eGFR) using CKD-EPI 2021 Creatinine mitigated the differences between groups and resulted in average values that were no longer significantly different between groups, with no patient showing eGFR higher than 140 mL/min/1.73 m^2^ in either of the groups (Table 2).

**Fig. 1:**
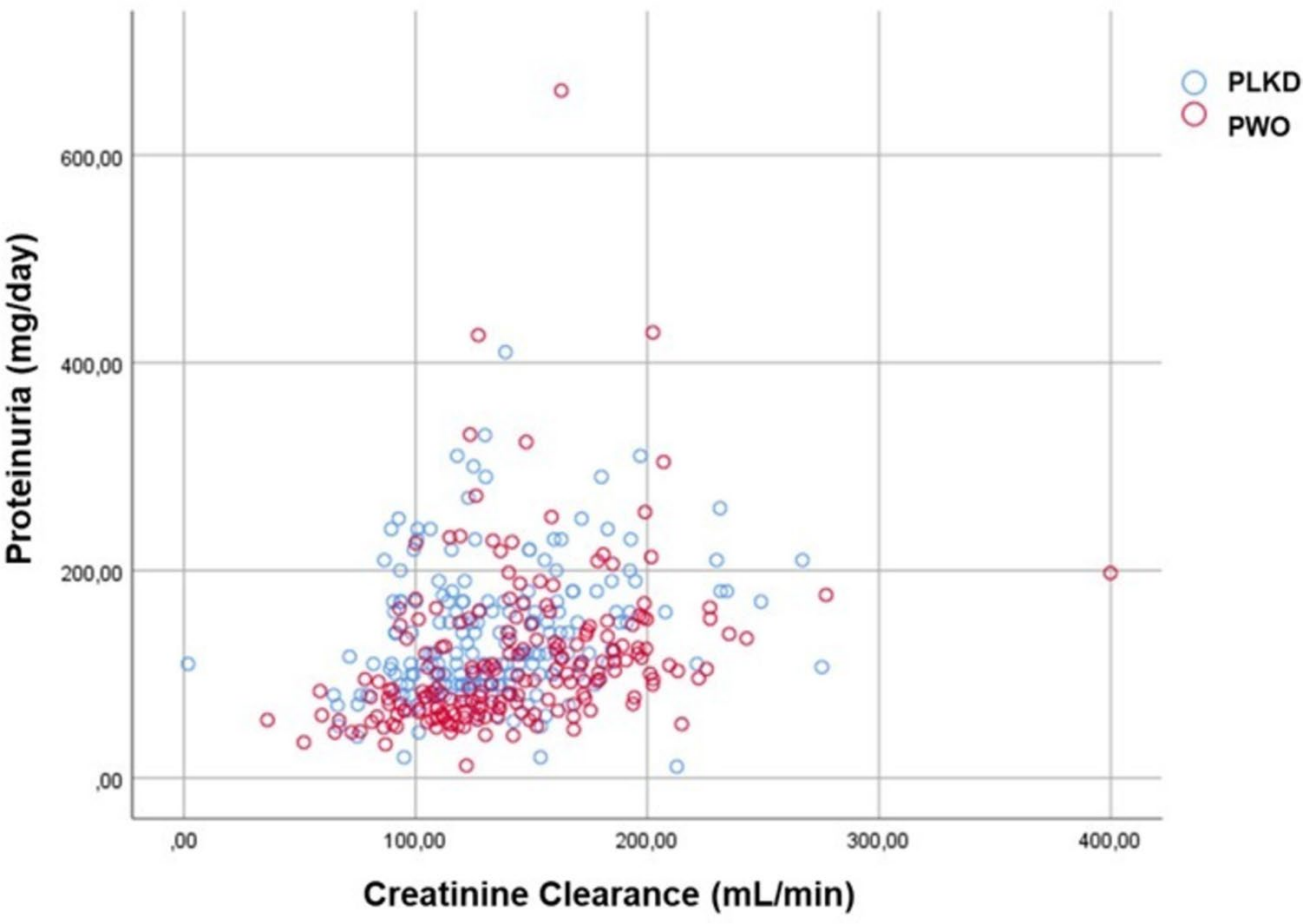
Distribution of patients according to the level of proteinuria and creatinine clearance (red circles, PWO; blue circles, PLKD)

Although average proteinuria levels were higher in PWO, 38.5% of PLKD presented proteinuria higher than 150 mg/day. This difference was made more evident after filtering for patients with proteinuria higher than 150 mg/day, which the average proteinuria was higher in PWO (329.58 ± 719.65 mg/day vs 199.18 ± 50.74 mg/day in PLKD). Figure 1 shows the distribution of patients according to proteinuria and creatinine clearance not corrected for BSA, documenting that for lower levels of creatinine clearance, in PLKD, there is a pattern of proteinuria that ranges within mild figures, while in PWO, higher proteinuria levels are linked to higher creatinine clearance levels.

Logistic regression using proteinuria either as a continuous variable (Table 3) or as a categoric variable (higher or lower than 150 mg/day) (Table 4) showed that creatinine clearance had a significant impact on proteinuria in PWO (HR 1.522, *p* < 0.05 and HR 1.014, *p* < 0.05, respectively). In PLKD, creatinine clearance also showed to impact proteinuria as a continuous variable, but with a lower strength (HR 0.376, *p* < 0.05) (Table 3), and as a categoric variable (higher or lower than 150 mg/day) (HR 1.015, *p* < 0.05) (Table 4).

**Table 3:** Univariate analysis using proteinuria as a continuous variable as an outcome

Proteinuria		
	Univariate	*p*-value
	HR (95% CI)	
**PLKD**		
Uric acid (mg/dL)	− 5.653 (− 13.640 to 2,334)	0.164
Hemoglobin (g/dL)	− 3.800 (− 11.316 to 3.716)	0.320
Glucose (mg/dL)	− 0.434 (− 1.328 to 0.461)	0.340
Total cholesterol (mg/dL)	0.006 (− 0.228 to 0.241)	0.959
Triglycerides (mg/dL)	− 0.061 (− 0.237 to 0.115)	0.496
HDL cholesterol (mg/dL)	− 0.098 (− 0.729 to 0.533)	0.760
LDL cholesterol (mg/dL)	0.118 (− 0.200 to 0.436)	0.465
Serum creatinine (mg/dL)	− 20.217 (− 76.885 to 36.451)	0.483
Age (years)	0.033 (− 0.824 to 0.890)	0.940
BMI (kg/m^2^)	1.746 (− 0.869 to 4.361)	0.190
CrCl/BSA (mL/min)	0.377 (0.138–0.617)	**0.002**
CrCl (mL/min)	0.376 (0.159–0.593)	**0.001**
eGFR (mL/min/1.73 m^2^)	0.077 (− 0.563 to 0.717)	0.812
**PWO**		
Uric acid (mg/dL)	− 6.143 (− 43.726 to 31.440)	0.748
Hemoglobin (g/dL)	4.621 (− 38.390 to 47.632)	0.832
Glucose (mg/dL)	− 1.197 (− 5.442 to 3.048)	0.579
Total cholesterol (mg/dL)	0.078 (− 1.294 to 1.451)	0.910
Triglycerides (mg/dL)	0.013 (− 0.783 to 0.809)	0.974
HDL cholesterol (mg/dL)	0.191 (− 4.348 to 4.729)	0.934
LDL cholesterol (mg/dL)	0.217 (− 1.150 to 1.583)	0.755
Serum creatinine (mg/dL)	− 205.075 (− 585.053 to 174.903)	0.288
Age (years)	− 2.816 (− 7.139 to 1.507)	0.200
BMI (kg/m^2^)	2.645 (− 6.654 to 11.944)	0.575
CrCl/BSA (mL/min)	1.887 (0.439–3.335)	**0.011**
CrCl (mL/min)	1.522 (0.455–2.589)	**0.005**
eGFR (mL/min/1.73 m^2^)	3.112 (− 0.376 to 6.599)	0.080

Abbreviations: *PWO* patient with obesity, *PLKD* potential living kidney donors, *BMI* body mass index, *HDL* high-density lipoprotein, *LDL* low-density lipoprotein, *CrCl* creatinine clearance, *BSA* body surface area, *eGFR* estimated glomerular filtration rate

Data in bold emphasis indicates a statistically significant p-value

**Table 4:** Univariate and multivariate analysis using proteinuria as a categoric variable (higher or lower than 140 mL/min) as an outcome

	Univariate		Multivariate	
	HR (95% CI)	*p*-value	HR (95% CI)	*p*-value
**PLKD**				
Uric acid (mg/dL)	0.795 (0.596–1.060)	0.795	–	-
Hemoglobin (g/dL)	0.898 (0.700–1.151)	0.396	–	-
Glucose (mg/dL)	0.977 (0.945–1.010)	0.167	–	-
Total cholesterol (mg/dL)	0.999 (0.992–1.007)	0.885	–	-
Triglycerides (mg/dL)	0.997 (0.991–1.003)	0.437	–	-
HDL cholesterol (mg/dL)	1.005 (0.985–1.026)	0.635	–	-
LDL cholesterol (mg/dL)	1.002 (0.992–1.012)	0.703	–	-
Serum creatinine (mg/dL)	0.139 (0.020–0.984)	**0.048**	0.294 (0.038–2.260)^$^	0.294^$^
			0.199 (0.027–1.465)^#^	0.133^#^
Age (years)	0.998 (0.971–1.027)	0.898	–	-
BMI (kg/m^2^)	1.066 (0.978–1.163)	0.144	–	-
CrCl/BSA (mL/min)	1.014 (1.005–1.023)	**0.001**	1.013 (1.004–1.022)^§^	**0.005**^§^
CrCl (mL/min)	1.015 (1.007–1.024)	**0.000**	1.015 (1.006–1.023)^§^	**0.001**^§^
eGFR (mL/min/1.73 m^2^)	1.011 (0.990–1.033)	0.313	–	-
**PWO**				
Uric acid (mg/dL)	1.186 (0.925–1.522)	0.179		
Hemoglobin (g/dL)	1.228 (0.921–1.637)	0.162		
Glucose (mg/dL)	1.014 (0.986–1.042)	0.340		
Total cholesterol (mg/dL)	1.002 (0.993–1.011)	0.657		
Triglycerides (mg/dL)	1.003 (0.998–1.009)	0.185		
HDL cholesterol (mg/dL)	0.973 (0.941–1.006)	0.104		
LDL cholesterol (mg/dL)	1.002 (0.993–1.011)	0.646		
Serum creatinine (mg/dL)	2.651 (0.242–28.997)	0.424		
Age (years)	1.003 (0.974–1.033)	0.821		
BMI (kg/m^2^)	1.025 (0.965–1.089)	0.417		
CrCl/BSA (mL/min)	1.019 (1.008–1.031)	**0.001**		
CrCl (mL/min)	1.014 (1.006–1.022)	**0.001**		
eGFR (mL/min/1.73 m^2^)	1.011 (0.986–1.036)	0.404		

^$^Adjusted to CrCl/BSA values

^#^Adjusted to CrCl levels

§Adjusted to serum creatinine levels

Abbreviations: *PWO* patient with obesity, *PLKD* potential living kidney donors, *BMI* body mass index, *HDL* high-density lipoprotein, *LDL* low-density lipoprotein, *CrCl* creatinine clearance, *BSA* body surface area, *eGFR* estimated glomerular filtration rate

Data in bold emphasis indicates a statistically significant p-value

ROC analysis showed creatinine clearance adjusted and unadjusted for BSA had a significant predictive capacity for proteinuria (AUC 0.630, *p* < 0.05 and AUC 0.660, *p* < 0.05, respectively), while BMI was not predictive for proteinuria level.

## Discussion

In this work, we sought to identify the biomarkers that characterize obesity-related kidney disease. For this, we compared a group of PWO candidates for bariatric surgery with a group of PLKD candidates without obesity, expectedly devoid of clinically relevant health conditions known to impact kidney function, and therefore used as a control.

Important methodological issues were considered. Firstly, patients were matched according to age and sex, two variables that are known to have a significant impact in glomerular filtration rate and proteinuria levels [22, 29]. Moreover, there are significant differences in body composition inherent to age and sex [23, 30], to which this pair-matching also answers. Secondly, measurements were based on a 24-h urine collection. Creatinine clearance was evaluated with and without indexing for BSA, to ascertain the impact of the systematic underestimation of glomerular filtration rate (GFR) that happens with BSA indexing, which affects particularly patients with obesity [17, 18]. Finally, the fact that patients with diabetes or any inflammatory disease were excluded from the study allowed us to reduce the chances of bias due to potential kidney alterations associated not with obesity but with diabetic kidney disease or inflammatory etiologies.

The proportion of individuals with creatinine clearance greater than 140 mL/min and average creatinine clearance were significantly higher in PWO compared to PLKD. Unsurprisingly, since obesity is characterized by increased renal plasma flow, glomerular filtration rate, and filtration fraction [31–33]. Noticeably, indexing creatinine clearance for BSA inverted these results: the proportion of patients with filtration rates greater than 140 mL/min was lower in PWO compared to PLKD and average creatinine clearance corrected for BSA was lower in PWO. Measurements of GFR such as creatinine clearance are commonly corrected for BSA to account for differences in body size. However, in PWO, this has shown to lead to a systematic underestimation of GFR [18, 34, 35], because GFR does not increase in parallel to adiposity, but is rather a result of single nephron filtration increase [17–19]. The higher the BMIs the more significant the masking effect of indexing creatinine clearance for BSA, resulting in a misleading creatinine clearance value, lower in PWO. Therefore, our study further reinforces the need to abandon the use of eGFR calculations that use standardization for BSA in the setting of obesity. Differences between measured creatinine clearance and eGFR calculated using CKD-EPI-Creatinine 2021 also became apparent in our study. Average values of eGFR were significantly below the value of measured creatinine clearance for both groups, mitigating the differences between groups. The CKD-EPI Creatinine equation is based on sex, age, and serum creatinine and has been shown to significantly overestimate the presence of chronic kidney disease and underestimate the rate of hyperfiltration in the population with obesity [36]. This has motivated to attempts to the development of new eGFR equation models to be specifically applied in PWO [37].

Average proteinuria was also significantly higher among PWO, a difference which was even more striking when only proteinuria greater than 150 mg/day was considered. Proteinuria is a characteristic feature of obesity-related glomerulopathy [2]. Additionally, adiposity was shown to correlate with protein excretion [38, 39]. Indeed, in a recent cross-sectional study with more than 400,000 people from the UK Biobank, for each BMI increment of 5 kg/m^2^, the odds of a higher albuminuria category were 47% greater [40]. Unexpectedly, although the magnitude proteinuria was lower, the number of individuals with clinically significant proteinuria was greater in PLKD (Table 2). Although this is not quite surprising, since low levels of proteinuria may be acceptable in PLDK, kidney donation in the presence of borderline medical abnormalities is being increasingly accepted [41–43]. Several factors have been appointed as the culprits for proteinuria in PWO [44]. One of the major determinants shown to impact on proteinuria is hyperfiltration [8, 45, 46]. Our logistic regression model showed that creatinine clearance positively impacted on the levels of proteinuria in PWO, but to a lower extent in PLKD (Table 3). This effect was observed even when creatinine clearance was corrected for BSA in both groups. This is a significant finding, as it suggests that in PWO hyperfiltration is disproportionately detrimental to the glomerular barrier, translating in greater proteinuria as compared to individuals of the control group with similar hyperfiltration, suggesting that in the presence of obesity, the mechanisms leading to proteinuria might differ. Thus, obesity may favor pathophysiological pathways that translate into glomerular protein loss in the setting of hyperfiltration. Indeed, besides modifying glomerular hemodynamics, several other obesity-specific kidney lesion pathways have been described [47, 48]. A detailed evaluation of the albumin-to-protein ratio [49–51] and proteomic analysis could be valuable to distinguish between glomerular versus tubular proteinuria and differentiate lesion pathways[52].

Of particular notice, neither cholesterol nor serum uric acid was shown to significantly affect proteinuria in any of the groups, in contrast to what other authors have shown [53, 54],

This study harbors some limitations to acknowledge. The cross-sectional nature of our study is an inherent limitation. The analysis would be greatly enriched with follow-up of these patients, possibly providing new insights about particularities in the pathophysiology of obesity-related kidney disease. Additionally, measurements of albuminuria and other urinary proteins could be valuable to better characterize kidney dysfunction and evaluate specific pathways of the lesion. Notwithstanding, our study has several important strengths. The fact that we used propensity analysis to minimize differences in baseline characteristics between both groups and had access to a large number of patients allowed us enough power to depict robust statistical differences. Furthermore, we evaluated kidney function using a 24-h urine collection and analyzed creatinine clearance using indexed and non-indexed values.

In conclusion, glomerular hyperfiltration and disproportionate proteinuria are the hallmarks of obesity-related kidney dysfunction. Given the prevalence of kidney disorders and the challenges of kidney function assessment in obesity, our study suggests that proteinuria should be routinely evaluated in bariatric surgery candidates.

## Data Availability

No datasets were generated or analysed during the current study.

## References

[1] Yau K, Kuah R, Cherney DZI, Lam TKT. Obesity and the kidney: mechanistic links and therapeutic advances. Nat Rev Endocrinol. 2024;20(6):321–35. 10.1038/s41574-024-00951-7. 38351406 10.1038/s41574-024-00951-7

[2] D’Agati VD, Chagnac A, de Vries AP, et al. Obesity-related glomerulopathy: clinical and pathologic characteristics and pathogenesis. Nat Rev Nephrol. 2016;12(8):453–71. 27263398 10.1038/nrneph.2016.75

[3] Bonnet F, Deprele C, Sassolas A, et al. Excessive body weight as a new independent risk factor for clinical and pathological progression in primary IgA nephritis. Am J Kidney Dis. 2001;37(4):720–7. 11273871 10.1016/s0272-6386(01)80120-7

[4] Griffin KA, Kramer H, Bidani AK. Adverse renal consequences of obesity. Am J Physiol Renal Physiol. 2008;294(4):F685-696. 18234955 10.1152/ajprenal.00324.2007

[5] Strazzullo P, Barba G, Cappuccio FP, et al. Altered renal sodium handling in men with abdominal adiposity: a link to hypertension. J Hypertens. 2001;19(12):2157–64. 11725158 10.1097/00004872-200112000-00007

[6] Engeli S, Böhnke J, Gorzelniak K, et al. Weight loss and the renin-angiotensin-aldosterone system. Hypertension. 2005;45(3):356–62. 15630041 10.1161/01.HYP.0000154361.47683.d3

[7] Tsuboi N, Okabayashi Y, Shimizu A, et al. The renal pathology of obesity. Kidney Int Rep. 2017;2(2):251–60. 29142961 10.1016/j.ekir.2017.01.007 PMC5678647

[8] de Vries AP, Ruggenenti P, Ruan XZ, et al. Fatty kidney: emerging role of ectopic lipid in obesity-related renal disease. Lancet Diabetes Endocrinol. 2014;2(5):417–26. 24795255 10.1016/S2213-8587(14)70065-8

[9] Ouchi N, Parker JL, Lugus JJ, et al. Adipokines in inflammation and metabolic disease. Nat Rev Immunol. 2011;11(2):85–97. 21252989 10.1038/nri2921 PMC3518031

[10] Virtue S, Vidal-Puig A. Adipose tissue expandability, lipotoxicity and the Metabolic Syndrome–an allostatic perspective. Biochim Biophys Acta. 2010;1801(3):338–49. 20056169 10.1016/j.bbalip.2009.12.006

[11] Simon N, Hertig A. Alteration of fatty acid oxidation in tubular epithelial cells: from acute kidney injury to renal fibrogenesis. Front Med (Lausanne). 2015;2:52. 26301223 10.3389/fmed.2015.00052 PMC4525064

[12] Chen Y, Deb DK, Fu X, et al. ATP-citrate lyase is an epigenetic regulator to promote obesity-related kidney injury. Faseb J. 2019;33(8):9602–15. 31150280 10.1096/fj.201900213R PMC6662982

[13] Jiang T, Wang Z, Proctor G, et al. Diet-induced obesity in C57BL/6J mice causes increased renal lipid accumulation and glomerulosclerosis via a sterol regulatory element-binding protein-1c-dependent pathway. J Biol Chem. 2005;280(37):32317–25. 16046411 10.1074/jbc.M500801200

[14] Dorotea D, Koya D, Ha H. Recent insights Into SREBP as a direct mediator of kidney fibrosis via lipid-independent pathways. Front Pharmacol. 2020;11:265. 32256356 10.3389/fphar.2020.00265 PMC7092724

[15] Zhu Q, Scherer PE. Immunologic and endocrine functions of adipose tissue: implications for kidney disease. Nat Rev Nephrol. 2018;14(2):105–20. 29199276 10.1038/nrneph.2017.157

[16] Wang TY, Wang RF, Bu ZY, et al. Association of metabolic dysfunction-associated fatty liver disease with kidney disease. Nat Rev Nephrol. 2022;18(4):259–68. 35013596 10.1038/s41581-021-00519-y

[17] Cachat F, Combescure C, Cauderay M, et al. A systematic review of glomerular hyperfiltration assessment and definition in the medical literature. Clin J Am Soc Nephrol. 2015;10(3):382–9. 25568216 10.2215/CJN.03080314 PMC4348676

[18] Delanaye P, Mariat C, Cavalier E, et al. Errors induced by indexing glomerular filtration rate for body surface area: reductio ad absurdum. Nephrol Dial Transplant. 2009;24(12):3593–6. 19734136 10.1093/ndt/gfp431

[19] López-Martínez M, Luis-Lima S, Morales E, et al. The estimation of GFR and the adjustment for BSA in overweight and obesity: a dreadful combination of two errors. Int J Obes (Lond). 2020;44(5):1129–40. 31641213 10.1038/s41366-019-0476-z

[20] Reese PP, Boudville N, Garg AX. Living kidney donation: outcomes, ethics, and uncertainty. Lancet. 2015;385(9981):2003–13. 26090646 10.1016/S0140-6736(14)62484-3

[21] Almeida M, Ribeiro C, Silvano J, Pedroso S, Tafulo S, Martins S, Ramos M, Malheiro J. Living donors’ age modifies the impact of pre-donation estimated glomerular filtration rate on graft survival. J Clin Med. 2023;12(21):6777. 10.3390/jcm12216777. 37959241 10.3390/jcm12216777 PMC10649187

[22] Stevens LA, Levey AS. Measured GFR as a confirmatory test for estimated GFR. J Am Soc Nephrol. 2009;20(11):2305–13. 19833901 10.1681/ASN.2009020171

[23] Kim SK, Kwon YH, Cho JH, et al. Changes in body composition according to age and sex among young non-diabetic Korean adults: the Kangbuk Samsung health study. Endocrinol Metab (Seoul). 2017;32(4):442–50. 29199402 10.3803/EnM.2017.32.4.442 PMC5744730

[24] Lentine KL, Kasiske BL, Levey AS, et al. Summary of kidney disease: improving global outcomes (KDIGO) clinical practice guideline on the evaluation and care of living kidney donors. Transplantation. 2017;101(8):1783–92. 28737659 10.1097/TP.0000000000001770 PMC5542788

[25] Manas D, Burnapp L, Andrews PA. Summary of the British Transplantation Society UK guidelines for living donor liver transplantation. Transplantation. 2016;100(6):1184–90. 26950721 10.1097/TP.0000000000001128

[26] Inker LA, Eneanya ND, Coresh J, et al. New creatinine- and cystatin c-based equations to estimate GFR without race. N Engl J Med. 2021;385(19):1737–49. 34554658 10.1056/NEJMoa2102953 PMC8822996

[27] Du Bois D, Du Bois EF. A formula to estimate the approximate surface area if height and weight be known. 1916. Nutrition. 1989;5(5):303–11 (**discussion 312-303**). 2520314

[28] Stevens PE, Levin A. Evaluation and management of chronic kidney disease: synopsis of the kidney disease: improving global outcomes 2012 clinical practice guideline. Ann Intern Med. 2013;158(11):825–30. 23732715 10.7326/0003-4819-158-11-201306040-00007

[29] Levey AS, Inker LA, Coresh J. GFR estimation: from physiology to public health. Am J Kidney Dis. 2014;63(5):820–34. 24485147 10.1053/j.ajkd.2013.12.006 PMC4001724

[30] Karvonen-Gutierrez C, Kim C. Association of mid-life changes in body size, body composition and obesity status with the menopausal transition. Healthcare (Basel). 2016;4(3):42. 10.3390/healthcare4030042. 27417630 10.3390/healthcare4030042 PMC5041043

[31] Porter LE, Hollenberg NK. Obesity, salt intake, and renal perfusion in healthy humans. Hypertension. 1998;32(1):144–8. 9674651 10.1161/01.hyp.32.1.144

[32] Reisin E, Messerli FG, Ventura HO, et al. Renal haemodynamic studies in obesity hypertension. J Hypertens. 1987;5(4):397–400. 3668243

[33] Chagnac A, Herman M, Zingerman B, et al. Obesity-induced glomerular hyperfiltration: its involvement in the pathogenesis of tubular sodium reabsorption. Nephrol Dial Transplant. 2008;23(12):3946–52. 18622024 10.1093/ndt/gfn379

[34] Slone TH. Body surface area misconceptions. Risk Anal. 1993;13(4):375–7. 8234944 10.1111/j.1539-6924.1993.tb00736.x

[35] Dooley MJ, Poole SG. Poor correlation between body surface area and glomerular filtration rate. Cancer Chemother Pharmacol. 2000;46(6):523–6. 11138467 10.1007/PL00006751

[36] Domislovic M, Domislovic V, Fucek M, et al. Should the CKD EPI equation be used for estimation of the glomerular filtration rate in obese subjects? Kidney Blood Press Res. 2022;47(10):597–604. 36170804 10.1159/000526115

[37] Basolo A, Salvetti G, Giannese D, et al. Obesity, hyperfiltration, and early kidney damage: a new formula for the estimation of creatinine clearance. J Clin Endocrinol Metab. 2023;108(12):3280–6. 37296533 10.1210/clinem/dgad330 PMC10655541

[38] Pehlivan E, Ozen G, Taskapan H, et al. Identifying the determinants of microalbuminuria in obese patients in primary care units: the effects of blood pressure, random plasma glucose and other risk factors. J Endocrinol Invest. 2016;39(1):73–82. 26093468 10.1007/s40618-015-0331-6

[39] Basdevant A, Cassuto D, Gibault T, et al. Microalbuminuria and body fat distribution in obese subjects. Int J Obes Relat Metab Disord. 1994;18(12):806–11. 7894519

[40] Zhu P, Lewington S, Haynes R, et al. Cross-sectional associations between central and general adiposity with albuminuria: observations from 400,000 people in UK Biobank. Int J Obes (Lond). 2020;44(11):2256–66. 32678323 10.1038/s41366-020-0642-3 PMC7577847

[41] Bellini MI, Nozdrin M, Pengel L, et al. How good is a living donor? Systematic review and meta-analysis of the effect of donor demographics on post kidney transplant outcomes. J Nephrol. 2022;35(3):807–20. 35072936 10.1007/s40620-021-01231-7 PMC8995249

[42] Reese PP, Caplan AL, Kesselheim AS, et al. Creating a medical, ethical, and legal framework for complex living kidney donors. Clin J Am Soc Nephrol. 2006;1(6):1148–53. 17699340 10.2215/CJN.02180606

[43] Reese PP, Feldman HI, McBride MA, et al. Substantial variation in the acceptance of medically complex live kidney donors across US renal transplant centers. Am J Transplant. 2008;8(10):2062–70. 18727695 10.1111/j.1600-6143.2008.02361.x PMC2590588

[44] Pereira PR, Pereira J, Braga PC, Pereira SS, Nora M, Guimarães M, Monteiro MP, Rodrigues A. Renal dysfunction phenotypes in patients undergoing obesity surgery. Biomolecules. 2023;13(5):790. 10.3390/biom13050790. 37238660 10.3390/biom13050790 PMC10216106

[45] Lee SM, Park JY, Park MS, et al. Association of renal hyperfiltration with incident proteinuria - a nationwide registry study. PLoS ONE. 2018;13(4):e0195784. 29652920 10.1371/journal.pone.0195784 PMC5898733

[46] Cortinovis M, Perico N, Ruggenenti P, et al. Glomerular hyperfiltration. Nat Rev Nephrol. 2022;18(7):435–51. 35365815 10.1038/s41581-022-00559-y

[47] Lazar MA. Resistin- and obesity-associated metabolic diseases. Horm Metab Res. 2007;39(10):710–6. 17952831 10.1055/s-2007-985897

[48] Bourebaba L, Marycz K. Pathophysiological implication of fetuin-a glycoprotein in the development of metabolic disorders: A Concise Review. J Clin Med. 2019;8(12):2033. 10.3390/jcm8122033. 31766373 10.3390/jcm8122033 PMC6947209

[49] Bökenkamp A. Proteinuria-take a closer look! Pediatr Nephrol. 2020;35(4):533–41. 31925536 10.1007/s00467-019-04454-w PMC7056687

[50] Hassan W, Shrestha P, Sumida K, et al. Association of uric acid-lowering therapy with incident chronic kidney disease. JAMA Netw Open. 2022;5(6):e2215878. 35657621 10.1001/jamanetworkopen.2022.15878 PMC9166229

[51] Wang K, Kestenbaum B. Proximal tubular secretory clearance: a neglected partner of kidney function. Clin J Am Soc Nephrol. 2018;13(8):1291–6. 29490976 10.2215/CJN.12001017 PMC6086711

[52] Lee SY, Choi ME. Urinary biomarkers for early diabetic nephropathy: beyond albuminuria. Pediatr Nephrol. 2015;30(7):1063–75. 25060761 10.1007/s00467-014-2888-2 PMC4305495

[53] Jalal DI, Rivard CJ, Johnson RJ, et al. Serum uric acid levels predict the development of albuminuria over 6 years in patients with type 1 diabetes: findings from the Coronary Artery Calcification in Type 1 Diabetes study. Nephrol Dial Transplant. 2010;25(6):1865–9. 20064950 10.1093/ndt/gfp740 PMC2902891

[54] Yamagata K, Ishida K, Sairenchi T, et al. Risk factors for chronic kidney disease in a community-based population: a 10-year follow-up study. Kidney Int. 2007;71(2):159–66. 17136030 10.1038/sj.ki.5002017

